# Trends in Gestational Weight Gain in Louisiana, March 2019 to March 2022

**DOI:** 10.1001/jamanetworkopen.2023.31277

**Published:** 2023-08-29

**Authors:** Emily W. Harville, Chelsea L. Kracht, Nicole L. Cohen, Elizabeth F. Sutton, Maryam Kebbe, Leanne M. Redman

**Affiliations:** 1 Department of Epidemiology, Tulane University School of Public Health and Tropical Medicine, New Orleans, Louisiana; 2 Pennington Biomedical Research Center, Baton Rouge, Louisiana; 3 Woman’s Hospital, Baton Rouge, Louisiana; 4 Faculty of Kinesiology, University of New Brunswick, Fredericton, New Brunswick, Canada

## Abstract

**Importance:**

Average gestational weight gain (GWG) increased during the COVID-19 pandemic, but it is not known whether this trend has continued.

**Objective:**

To examine patterns of GWG during the COVID-19 pandemic by delivery and conception timing through the second year of the pandemic.

**Design, Setting, and Participants:**

This cohort study is a retrospective review of birth certificate and delivery records from 2019 to 2022. Electronic health records were from the largest delivery hospital in Louisiana. Participants included all individuals giving birth from March 2019 to March 2022. Data analysis was performed from October 2022 to July 2023.

**Exposure:**

Delivery date (cross-sectionally) and conception before the pandemic (March 2019 to March 2020) and during the peak pandemic (March 2020 to March 2021) and late pandemic (March 2021 to March 2022).

**Main Outcomes and Measures:**

The primary outcome was GWG (total GWG and adherence to the 2009 Institute of Medicine recommendations) analyzed using linear and log-linear regression with control for covariates.

**Results:**

Among 23 012 total deliveries (8763 Black individuals [38.1%]; 11 774 White individuals [51.2%]; mean [SD] maternal age, 28.9 [5.6] years), 3182 individuals (42.0%) exceeded the recommended weight gain in the year proceeding the pandemic, 3400 (45.4%) exceeded recommendations during the peak pandemic, and 3273 (44.0%) exceeded recommendations in the late pandemic. Compared with those who delivered before the pandemic (reference), participants had higher total GWG if they delivered peak or late pandemic (adjusted β [SE], 0.38 [0.12] kg vs 0.19 [0.12] kg; *P* = .007). When cohorts were defined by conception date, participants who conceived before the pandemic but delivered after the pandemic started had higher GWG compared with those whose entire pregnancy occurred before the pandemic (adjusted β [SE], 0.51 [0.16] kg). GWG was lower in the pregnancies conceived after the pandemic started and the late pandemic (adjusted β [SE], 0.29 [0.12] kg vs 0.003 [0.14] kg; *P* = .003) but these participants began pregnancy at a slightly higher weight. Examining mean GWG month by month suggested a small decrease for March 2020, followed by increased mean GWG for the following year. Individuals with 2 pregnancies (1289 individuals) were less likely to gain weight above the recommended guidelines compared with their prepandemic pregnancy, but this association was attenuated after adjustment.

**Conclusions and Relevance:**

In this cohort, individuals with critical time points of their pregnancy during the COVID-19 pandemic gained more weight compared with the previous year. The increased GWG leveled off as the pandemic progressed but individuals were slightly heavier beginning pregnancy.

## Key Points

**Question:**

Was the COVID-19 pandemic associated with changes in patterns of gestational weight gain?

**Findings:**

In this cohort study using electronic medical records for 23 012 deliveries, gestational weight gain was close to prepandemic patterns in later pandemic deliveries (March 13, 2021, to March 12, 2022) but individuals began pregnancy slightly heavier than before the pandemic.

**Meaning:**

These findings suggest that gestational weight gain has plateaued in the later parts of the pandemic, although individuals are beginning pregnancy at a higher weight than before the pandemic.

## Teaser

This cohort study examines patterns of gestational weight gain during the COVID-19 pandemic by delivery and conception timing through the second year of the pandemic.

## Introduction

Appropriate weight gain during pregnancy is critical for maternal and child health, yet only one-third of women achieve this goal.^1^ Most pregnant individuals experience excess gestational weight gain (GWG), which may have harmful consequences.^2,3,4^ including exacerbated risk for cardiovascular disease.^5^ Even within the same individual, every additional 2 kg gained beyond GWG guidelines increases the risk for macrosomia for the child,^6^ which is related to lifelong risk for obesity.^7^ Furthermore, individuals with overweight and obesity are at higher risk for exceeding GWG guidelines, because they are recommended to gain less weight during pregnancy compared with individuals with normal weight.^1,7^

The COVID-19 outbreak led to community social distancing, while medical centers enacted policies to reduce infectious disease spread.^8^ Accordingly, many people reported more time spent sedentary, unfavorable changes to eating behaviors, and worsened anxiety and depression.^9,10^ The pandemic had direct and indirect effects on pregnant individuals’ everyday lives, including higher depression,^11^ fewer prenatal and postpartum visits,^12,13^ and increased early-pregnancy adverse outcomes.^14^ These observations mirror disaster research,^15,16^ which suggests that exposure to disasters during pregnancy leads to increased incidence of pregnancy loss, birth defects, and adverse child development. Consideration of GWG in past disasters primarily centered on disruption to food supply and malnutrition.^17,18^ Although food insecurity increased in certain groups,^19^ calorie availability remained abundant in the US over the COVID-19 pandemic, which may lead to a different GWG pattern.^20^

Pregnancy cohorts from China and the US documented higher GWG during the first year of COVID-19 pandemic compared with those who delivered the prior year.^21,22,23^ The COVID-19 pandemic emergency phase continued for multiple years, but as vaccines became readily available, social distancing protocols loosened and scheduling of medical visits rebounded.^12^ Whether pregnancy-related outcomes have returned to prepandemic levels is not known. Considering changes in outcomes between pregnancies to the same individual may better demonstrate how changes in the environment impact these outcomes.^6^ Given the length of the COVID-19 pandemic for multiple years, it is now possible to examine multiple pregnancies, because typical birth spacing is between 2 and 3 years.^24^

To fill this gap, we examined patterns (consistency and direction) of GWG by delivery and conception in annual increments through the second year of the COVID-19 pandemic and explored changes in GWG within individuals who had pregnancies before and during the pandemic. We tested the hypothesis that fewer individuals with pandemic pregnancies would gain weight within GWG recommendations (mainly exceeding GWG recommendations) compared with those with a prepandemic pregnancy. We also hypothesized that individuals with a prepandemic and pandemic pregnancy would have higher GWG in their pandemic pregnancy.

## Methods

### Study Design

This cohort study is a retrospective examination of electronic health records and includes female individuals (aged 12-50 years) who delivered between January 1, 2017, and July 31, 2022, at a large women’s specialty hospital in Baton Rouge, Louisiana. Pandemic-affected pregnancies are considered those after March 13, 2020, the first COVID-19 case in Louisiana.^8^

This study follows the Strengthening the Reporting of Observational Studies in Epidemiology (STROBE) reporting guidelines for observational studies. This medical record review of deidentified data was determined to be exempt by Woman’s Hospital Foundation institutional review board and, thus, did not require informed consent documentation, in accordance with 45 CFR §46.

Data were abstracted from hospital delivery records using recommended practices for abstracting medical records^25^ and were linked to birth certificates. Structured Query Language programming using *International Statistical Classification of Diseases and Related Health Problems, Tenth Revision* diagnostic and procedure codes, admitting and discharge data, and nurse and physician record notes were used to compile data. The data set was validated for quality control by comparison with individual records and examination for outliers.

### Definition of Study Periods: COVID-19 Pandemic

GWG was defined by delivery date and estimated conception date for both aims.^26^ Analysis by delivery date only reflects cross-sectional, clinical experience across time, but will produce bias in trends for any condition that affects gestational age at birth, is affected strongly by gestational age of exposure, or has seasonal patterning.^26^ Three delivery date groups were created on the basis of calendar year: prepandemic (March 13, 2019, to March 12, 2020), peak pandemic (March 13, 2020, to March 12, 2021), and late pandemic (March 13, 2021, to March 12, 2022). Defining cohorts by conception date ensures comparable exposure timing and follow-up. Month of conception was estimated according to delivery date and gestational age (days) at delivery. Four groups were compared: prepandemic (conceived >10 months before the pandemic, June 1, 2018, to May 1, 2019), partial early pandemic (conceived before the pandemic but part of pregnancy occurred during the pandemic, October 1, 2019, to February 28, 2020), partial peak pandemic (conceived after the start of the pandemic and part of pregnancy was during the pandemic peak, March 1, 2020, to February 28, 2021), and late pandemic (conceived after restrictions were lifted, March 1, 2021, to September 1, 2021). Individuals with probable conception between May and October 2019 were not included in the analysis because pandemic exposure would vary by length of gestation (681 individuals). Months of delivery and conception were also analyzed as discrete and continuous variables, from March 2019 to March 2022.

### Gestational Weight Gain

Total GWG was calculated from routinely obtained self-reported weight measures reported at delivery (delivery weight minus prepregnancy weight). Body mass index (BMI) was calculated using prepregnancy weight and height using the standard equation (weight in kilograms divided by height in meters squared) and was classified as underweight (<18.5), normal weight (18.5-24.9), overweight (25-29.9), and obesity (≥30). GWG per week was calculated as total GWG divided by length of gestation (kilograms per weeks).^27^ Total GWG was compared with the Institute of Medicine Guidelines for GWG according to prepregnancy BMI category and singleton or multiple births (eTable 1 in Supplement 1). GWG below the recommended range was categorized as under, within the range was categorized as recommended, and above the range was categorized as above.^28^ Because most of the changes appeared to be in the above recommendations group, the recommended and under groups were combined for most analyses.

### Covariates

We examined covariates that were associated with the outcome in previous studies.^29,30^ Next, we determined whether their distribution varied across the pandemic in this sample, including the pregnant individual’s age, education level, participant-reported race and ethnicity, marital status, type of insurance, employment status, smoking, alcohol use, and parity. Race and ethnicity were analyzed in this study because of their previously reported associations with GWG.

### Statistical Analysis

Our goal was to detect an odds ratio of greater than or equal to 0.91 when comparing above vs recommended GWG between prepandemic and pandemic pregnancies (80% power). From prepandemic estimates,^1^ we sought 6000 prepandemic and 12 000 pandemic deliveries (α = .05). Participants with complete data were included in the analysis. The analysis included participants of American Indian, Asian, Black, Hispanic, or White race; participants with missing data on parity (6 participants), education (53 participants), or race (or reported as *other*, 542 participants) were excluded. Of the possible 26 807 records, the population missing data on GWG or covariates (974 participants [3.6%]) did not differ according to timing of delivery relative to the pandemic, but those individuals were more likely to be younger than 18 years, multiparous, Hispanic, or have less than a high school education.

For the first aim of this study, GWG metrics (total GWG, weight gain per week, and prepregnancy weight), and proportions in each GWG recommendation (under, recommended, or above) among pandemic groups were compared using 1-way analysis of variance and χ^2^ tests. Lack of independence due to multiple and repeat births was accounted for with generalized estimating equations with an exchangeable working correlation matrix. GWG and BMI are correlated, and an overall change in weight gain patterns could be associated with changes in prepregnancy BMI.^31^ Thus, interactions with prepregnancy BMI were tested, and models were further stratified by prepregnancy BMI. Finally, log-linear regression was used to compare gaining above vs recommended or under GWG guidelines (2 categories combined). To capture the magnitude above guidelines, we examined the total GWG above guidelines and percentage of participants who gained greater than or equal to 200% of the recommended GWG.

For month-by-month analyses, GWG was examined for each month between March 2019 and March 2022. A linear model with categorical terms for month, adjusted for covariates and accounting for correlation within each individual, was fit. Next, polynomial models with continuous time up to the quintic term were modeled. The *P* value and quasi-likelihood under the independence model criterion for the polynomial and the interaction terms were examined,^32^ which led to retaining the BMI interaction; the degree of the best-fitting polynomial varied from linear to quintic depending on BMI group (eFigure 1 and eFigure 2 in Supplement 1).

For the second aim, comparing participants with both a prepandemic and pandemic pregnancy, a dichotomous outcome was used (above GWG guidelines vs GWG recommended or under guidelines) for their GWG during each pregnancy. Discrepancies between pregnancies were analyzed using the McNemar test, and the timing of the pandemic (categories from aim 1) was used as a variable in conditional logistic regression models. These models were adjusted for covariates that varied between the 2 pregnancies: age and parity. For comparison, we examined 1374 individuals with 2 prepandemic pregnancies. All analyses were conducted in SAS statistical software version 9.4 (SAS Institute). Data analysis was performed from October 2022 to July 2023.

## Results

### Sample Description

The final analytic sample included 25 237 participants with either a delivery or pregnancy during the period. Among 23 012 deliveries, the mean (SD) maternal age was 28.9 (5.6) years, mean (SD) prepregnancy weight was 75.1 (21.0) kg, and most participants were either Black (8763 individuals [38.1%]) or White (11 774 individuals [51.2%]); 3182 participants (42.0%) exceeded the recommended weight gain in the year proceeding the pandemic, 3400 (45.4%) exceeded recommendations during peak pandemic, and 3273 (44.0%) exceeded in the late pandemic (Table 1). Across time periods, there were small shifts in maternal education, smoking status, alcohol use, and parity. Most individuals had overweight (5742 individuals [25.5%]) or obesity (7520 individuals [33.4%]) before pregnancy, although the largest BMI category was normal weight (8596 individuals [38.2%]). Current smoking was reported as less common (range, 2.9%-4.2%), and a greater proportion of births across the pandemic were to nulliparous individuals (range, 38.3%-41.0%).

**Table 1.** Descriptive Statistics of the Population Giving Birth Before and During the COVID-19 Pandemic by Delivery Date^a^

Variable	Participants, No. (%)				
	Total (N = 23 012)	Prepandemic (n = 7787)	Peak pandemic (n = 7671)	Late pandemic (n = 7554)	*P* value^b^
Prepregnancy BMI category^c^					
Underweight (<18.5)	643 (2.9)	209 (2.8)	211 (2.8)	223 (3.0)	.16
Normal (18.5-24.9)	8596 (38.2)	2956 (39.1)	2852 (38.1)	2788 (37.5)	
Overweight (25-29.9)	5742 (25.5)	1898 (25.1)	1970 (26.3)	1874 (25.2)	
Obesity (≥30)	7520 (33.4)	2506 (33.1)	2455 (32.8)	2559 (34.4)	
Age, y					
<18	222 (1.0)	68 (0.9)	79 (1.0)	75 (1.0)	.48
18-24	5199 (22.6)	1734 (22.3)	1769 (23.1)	1696 (22.5)	
25-29	7079 (30.8)	2460 (31.6)	2340 (30.5)	2279 (30.2)	
30-34	6617 (28.8)	2236 (28.7)	2158 (28.1)	2223 (29.4)	
35-39	3236 (14.1)	1082 (13.9)	1092 (14.2)	1062 (14.1)	
≥40	659 (2.9)	207 (2.7)	233 (3.0)	219 (2.9)	
Race and ethnicity					
Alaska Native or Pacific Islander	22 (0.10)	4 (0.05)	12 (0.16)	6 (0.08)	.11
Asian	495 (2.2)	169 (2.2)	160 (2.1)	166 (2.2)	
Black	8763 (38.1)	2972 (38.2)	2945 (38.4)	2846 (37.7)	
Hispanic	1958 (8.5)	650 (8.4)	612 (8.0)	696 (9.2)	
White	11 774 (51.2)	3992 (51.3)	3942 (51.4)	3840 (50.8)	
Marital status					
Married	11 584 (50.3)	3982 (51.1)	3880 (50.6)	3722 (49.3)	.06
Nonmarried	11 428 (49.7)	3805 (48.9)	3791 (49.4)	3832 (50.7)	
Education					
<12 y	2481 (10.8)	906 (11.6)	817 (10.7)	758 (10.0)	.001
High school	5877 (25.5)	1862 (23.9)	2028 (26.4)	1987 (26.3)	
Vocation school	1037 (4.5)	346 (4.4)	354 (4.6)	337 (4.5)	
Some college	4129 (17.9)	1460 (18.8)	1342 (17.5)	1327 (17.6)	
College	7065 (30.7)	2424 (31.1)	2314 (30.2)	2327 (30.8)	
Advanced degree	2423 (10.5)	789 (10.1)	816 (10.6)	818 (10.8)	
Employment					
Employed	13 479 (59.0)	4630 (59.8)	4424 (58.1)	4425 (59.0)	.12
Not employed	9383 (41.0)	3118 (40.2)	3189 (41.9)	3076 (41.0)	
Insurance					
Private	12 206 (53.1)	4182 (53.7)	4084 (53.3)	3940 (52.3)	.17
Medicaid, incarcerated, or self	10 771 (46.9)	3600 (46.3)	3574 (46.7)	3597 (47.7)	
Smoking					
Current	841 (3.7)	328 (4.2)	296 (3.9)	217 (2.9)	<.001
Former	1395 (6.1)	499 (6.4)	484 (6.3)	412 (5.5)	
Never	20 683 (90.2)	6936 (89.4)	6862 (89.8)	6885 (91.6)	
Alcohol use					
Yes	922 (4.08)	344 (4.5)	343 (4.6)	235 (3.2)	<.001
No	21 683 (95.9)	7338 (95.5)	7186 (95.4)	7159 (96.8)	
Parity					
0	9224 (40.1)	2982 (38.3)	3148 (41.0)	3094 (41.0)	.007
1	7201 (31.3)	2530 (32.5)	2331 (30.4)	2340 (31.0)	
2	4009 (17.4)	1398 (18.0)	1336 (17.4)	1275 (16.9)	
≥3	2578 (11.2)	877 (11.3)	856 (11.2)	845 (11.2)	

Abbreviation: BMI, body mass index.

^a^ Total number covers all deliveries and conceptions between March 13, 2019, and March 12, 2022. The sum of deliveries does not add to total because of conceptions occurring outside the listed time frames. Numbers may not sum to column total due to missing data on covariates. The prepandemic period is March 13, 2019, to March 12, 2020; the peak pandemic period is March 13, 2020, to March 12, 2021; and the late pandemic period is March 13, 2021, to March 12, 2022.

^b^ *P* values were assessed using χ^2^ analysis or Fisher exact test.

^c^ BMI is calculated as weight in kilograms divided by height in meters squared.

### GWG by Time Periods Defined by Delivery Date

Peak and late pandemic deliveries reported a slightly higher amount of total GWG and weight gain per week compared with prepandemic deliveries (Table 2). Compared with before the pandemic, participants were more likely to gain above GWG recommendations if they delivered during peak or late pandemic. Correspondingly, there was a lower proportion who gained under the GWG recommendations in peak and late pandemic.

**Table 2.** Differences in GWG by Pandemic Period and Delivery Date (N = 23 012)^a^

Variable	Prepandemic (n = 7787)	Peak pandemic (n = 7671)	Late pandemic (n = 7554)	*P* value^b^
GWG recommendations, participants, No. (%)				
Under	2169 (28.7)	1898 (25.4)	2061 (27.7)	<.001
Recommended	2218 (29.3)	2190 (29.3)	2110 (28.3)	
Above	3182 (42.0)	3400 (45.4)	3273 (44.0)	
Total unadjusted GWG, mean (SD), kg	12.5 (7.7)	12.9 (7.6)	12.8 (7.9)	.001
Adjusted GWG, β (SE)^c^				
Overall	1 [Reference]	0.38 (0.12)	0.19 (0.12)	.007
Stratified by BMI^d^				
<18.5	1 [Reference]	0.62 (1.94)	−0.66 (2.30)	.51
18.5-24.9	1 [Reference]	0.13 (0.16)	0.10 (0.16)	.70
25-29.9	1 [Reference]	0.47 (0.23)	0.61 (0.23)	.03
≥30	1 [Reference]	0.61 (0.23)	0.40 (0.23)	.03
Weight gain				
Unadjusted, mean (SD), kg/wk	0.32 (0.20)	0.34 (0.20)	0.33 (0.21)	<.001
Adjusted, β (SE)^c^				
Overall	1 [Reference]	0.01 (0.003)	0.01 (0.003)	.005
Stratified by BMI^d^				
<18.5	1 [Reference]	0.04 (0.05)	0.02 (0.06)	.50
18.5-24.9	1 [Reference]	0.003 (0.004)	0.003 (0.004)	.65
25-29.9	1 [Reference]	0.01 (0.01)	0.02 (0.01)	.02
≥30	1 [Reference]	0.02 (0.01)	0.01 (0.01)	.02
Prepregnancy weight				
Unadjusted, mean (SD), kg	74.7 (20.8)	74.8 (21.0)	75.4 (21.3)	.08
Adjusted, β (SE)^c^				
Overall	1 [Reference]	1.04 (0.46)	1.93 (0.65)	.02
Stratified by BMI^d^				
<18.5	1 [Reference]	−0.98 (0.87)	0.49 (1.47)	.14
18.5-24.9	1 [Reference]	−0.01 (0.15)	0.51 (0.14)	<.001
25-29.9	1 [Reference]	0.24 (0.22)	0.46 (0.21)	.09
≥30	1 [Reference]	1.42 (0.92)	3.72 (1.15)	.01

Abbreviations: BMI, body mass index; GWG, gestational weight gain.

^a^ The prepandemic period is March 13, 2019, to March 12, 2020; the peak pandemic period is March 13, 2020, to March 12, 2021; and the late pandemic period is March 13, 2021, to March 12, 2022.

^b^ Comparisons across all groups were assessed using analysis of variance (continuous outcome) or χ^2^ test (categorical outcome).

^c^ β values were calculated from generalized estimating equations (linear regression) adjusted for maternal age, parity, race and ethnicity, education, and marital status. Data on prepregnancy BMI were missing for 71 participants. Multiples were included in the analysis (n = 973).

^d^ BMI is calculated as weight in kilograms divided by height in meters squared.

As shown in Table 2, individuals who delivered during the peak and late pandemic had higher GWG than the prepandemic individuals (mean [SE] β, 0.38 [0.12] kg vs 0.19 [0.12] kg; *P* = .007). Individuals who delivered during peak and late pandemic were more likely to gain above GWG recommendations compared with prepandemic deliveries (adjusted rate ratio, 1.07 [95% CI, 3.00-10.00] vs 1.03 [95% CI, 1.00-1.07]). Individuals with overweight and obesity gained more GWG and weight gain per week at peak and late pandemic compared with their prepandemic counterparts. Individuals with normal weight who delivered during the late pandemic and those with obesity who delivered during the peak and late pandemic had a higher prepregnancy weight than their prepandemic counterparts. This amount translates to approximately 0.82 kg per person on average but 1.40 to 2.40 kg per person for individuals with obesity. The proportion who gained greater than or equal to 200% of guidelines increased across the pandemic (before the pandemic, 531 individuals [7.2%]; peak pandemic, 553 individuals [8.0%]; and late pandemic, 554 individuals [9.3%]), and the same pattern was seen in individuals with obesity (before the pandemic, 316 individuals [13.0%]; peak pandemic, 338 individuals [15.2%]; and late pandemic, 345 individuals [16.9%]) (eTable 2 in Supplement 1).

### GWG by Time Periods Defined by Estimated Conception Dates

Like the analysis by delivery date, there was a significant difference in all GWG metrics between estimated date of conception groups (Table 3). The partial early (1301 individuals [45.7%]) and partial peak pandemic (3300 individuals [44.8%]) groups reported the highest proportion above GWG guidelines compared with those with a prepandemic (3166 individuals [41.8%]) or late pandemic (1738 individuals [43.0%]) by estimated date of conception.

**Table 3.** Differences in GWG by Period by Estimated Conception Date (N = 22 331)^a^

Variable	Prepandemic (n = 7794)	Partial early pandemic (n = 2903)	Partial peak pandemic (n = 7500)	Late pandemic (n = 4134)	*P* value^b^
Unadjusted comparisons, mean (SD)					
Total GWG, kg	12.47 (7.67)	13.06 (7.75)	12.88 (7.84)	12.5 (7.84)	<.001
Weight gain, kg/wk	0.32 (0.20)	0.34 (0.20)	0.34 (0.20)	0.33 (0.20)	<.001
Prepregnancy weight, kg	74.67 (20.85)	75.07 (21.41)	75.21 (20.01)	75.83 (21.37)	.04
GWG guidelines, No. (%), participants					
Under	2166 (28.6)	731 (25.7)	1965 (26.7)	1125 (27.9)	.002
Recommended	2244 (29.6)	814 (28.6)	2108 (28.6)	1177 (29.1)	
Above	3166 (41.8)	1301 (45.7)	3300 (44.8)	1738 (43.0)	
Adjusted models, β (SE)^c^					
Total GWG, kg					
Overall	1 [Reference]	0.51 (0.16)	0.29 (0.12)	0.003 (0.14)	.003
Stratified by BMI^d^					
<18.5	1 [Reference]	1.23 (2.46)	−1.12 (1.87)	0.74 (3.69)	.48
18.5-24.9	1 [Reference]	0.33 (0.22)	0.30 (0.16)	−0.56 (0.19)	<.001
25-29.9	1 [Reference]	0.52 (0.31)	0.50 (0.23)	0.66 (0.28)	.06
≥30	1 [Reference]	0.75 (0.32)	0.47 (0.23)	0.72 (0.27)	.02
Weight gain, kg/wk					
Overall	1 [Reference]	0.01 (0.004)	0.01 (0.003)	0.001 (0.004)	<.001
Stratified by BMI^d^					
<18.5	1 [Reference]	0.06 (0.06)	0.003 (0.05)	0.05 (0.10)	.40
18.5-24.9	1 [Reference]	0.01 (0.01)	0.01 (0.004)	−0.01 (0.01)	<.001
25-29.9	1 [Reference]	0.01 (0.01)	0.01 (0.01)	0.02 (0.01)	.04
≥30	1 [Reference]	0.02 (0.01)	0.01 (0.01)	0.02 (0.01)	.02
Prepregnancy weight, kg					
Overall	1 [Reference]	0.18 (0.40)	1.18 (0.29)	1.21 (0.33)	<.001
Stratified by BMI					
<18.5	1 [Reference]	−1.71 (1.16)	−0.92 (1.11)	−1.34 (1.81)	.51
18.5-24.9	1 [Reference]	−0.001 (0.20)	0.34 (0.15)	0.63 (0.17)	<.001
25-29.9	1 [Reference]	−0.09 (0.28)	0.47 (0.22)	0.14 (0.26)	.11
≥30	1 [Reference]	1.63 (1.10)	3.00 (0.96)	2.32 (1.25)	.02

Abbreviations: BMI, body mass index; GWG, gestational weight gain.

^a^ The prepandemic period is June 1, 2018, to May 1, 2019; the partial early pandemic period is October 1, 2019, to February 28, 2020; the partial peak pandemic period is March 1, 2020, to February 28, 2021; and the late pandemic period is March 1, 2021, to September 1, 2021.

^b^ Comparisons across groups were assessed using analysis of variance (continuous outcome) or χ^2^ test (categorical outcome).

^c^ β values were calculated from generalized estimating equation (linear regression) adjusted for maternal age, parity, race and ethnicity, education, and marital status. Multiples included in analysis (n = 950). Data on prepregnancy BMI were missing for 86 participants.

^d^ BMI is calculated as weight in kilograms divided by height in meters squared.

Participants who conceived in partial early, partial peak, and late pandemic were 8%, 5%, and 3% more likely, respectively, to exceed the recommended weight gain compared with prepandemic pregnancies. Compared with prepandemic individuals, those who conceived during the partial early phase (mean β [SE], 0.51 [0.16] kg), partial peak phase (mean β [SE], 0.29 [0.12] kg), and late pandemic (mean β [SE], 0.003 [0.14] kg) reported more GWG (*P* = .003). Individuals with normal weight, overweight, and obesity had a higher total GWG and weight gain per week compared with their prepandemic counterparts. Individuals with normal weight and obesity had a higher prepregnancy weight when they conceived partial peak and late pandemic compared with prepandemic individuals.

### GWG by Month

Mean GWG varied by month, although absolute differences were small (Figure); patterns were similar by delivery or conception. Overall, there was lower mean GWG for those who delivered in March 2020. Compared with March 2019, individuals with normal weight who conceived in June 2019 (estimated March 2020 delivery) had lower mean GWG (adjusted β [SE], −1.43 [0.59] kg), as did those who conceived in March 2021 (adjusted β [SE], −1.54 [0.63] kg) and delivered in December 2021. Individuals with obesity who conceived in June 2019 had a lower mean weight gain (adjusted β [SE], −2.20 [0.86] kg) compared with those who conceived in March 2019. The polynomial model differed in degree by prepregnancy BMI category but showed flat and smooth patterns over time (eFigure 1 and eFigure 2 in Supplement 1).

**Figure.**
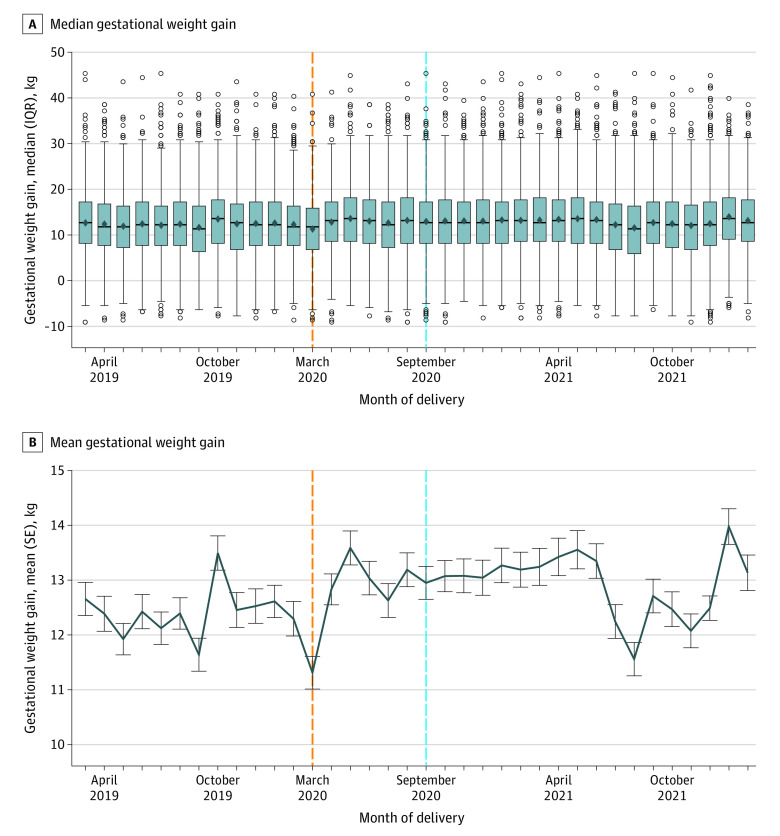
Gestational Weight Gain by Month of Delivery A, Median (IQR) gestational weight gain. Diamonds denote means, lines denote medians, boxes denote IQRs, circles denote outliers (>1.5 times the IQR), and error bars denote 95% CIs. B, Mean (SE) gestational weight gain. Error bars denote SEs, and points where lines intersect error bars denote means. Orange lines mark the start of the pandemic (March 2020), and the light blue lines indicate the start of pregnancies conceived and delivered after the pandemic or exposed to the pandemic during at least part of the pregnancy.

### Paired Interpregnancy Analyses

In paired analysis of 1289 individuals, the majority had similar GWG in both pregnancies (911 individuals [70.7%]), although approximately one-sixth (210 individuals [16.3%]) gained above guidelines in the prepandemic pregnancy but not in their pandemic pregnancy, and for 13.0% (168 individuals) the reverse occurred. More individuals who gained recommended or under GWG guidelines for both pregnancies received Medicaid or other insurance (310 individuals [52.6%]) compared with those who gained above GWG recommendations for both pregnancies (137 individuals [42.6%]). Within the discrepant experience group (378 individuals), those who gained recommended or under GWG guidelines in the prepandemic pregnancy and above the guidelines in postpandemic pregnancy had a higher prepregnancy BMI before the pandemic (mean [SE], 30.5 [8.0] kg) compared with the converse (prepandemic GWG above the guidelines and postpandemic GWG at recommended or under guidelines, mean [SE], 27.4 [6.9] kg). In the second pregnancy, this trend was reversed, with those who gained recommended or under having a slightly higher mean prepregnancy BMI (eTable 3 in Supplement 1).

Compared with their prepandemic pregnancy, participants were less likely to gain above GWG guidelines in the pandemic pregnancy, but after adjusting for covariates there was no association (Table 4). Those who conceived in the early pandemic had lower odds of gaining above guidelines compared with their prepandemic pregnancy, but after adjustment there was no difference. Similarly, individuals with 2 prepandemic pregnancies were not more likely to gain above GWG guidelines in their later pregnancy (adjusted odds ratio, 0.95; 95% CI, 0.53-1.71).

**Table 4.** Associations of Prepandemic and Postpandemic Pregnancies With Gaining Above Gestational Weight Gain Guidelines (N = 1289)^a^

Variable	OR (95% CI)	Adjusted OR (95% CI)
Overall		
Prepandemic	1 [Reference]	1 [Reference]
Postpandemic	0.80 (0.65-0.98)	0.89 (0.51-1.56)
Delivery date		
Prepandemic	1 [Reference]	1 [Reference]
Early pandemic	0.73 (0.52-1.03)	0.85 (0.51-1.42)
Late pandemic	0.78 (0.59-1.04)	0.97 (0.50-1.89)
Conception		
Prepandemic	1 [Reference]	1 [Reference]
Partial early pandemic	0.57 (0.33-0.97)	0.6 6 (0.34-1.26)
Partial peak pandemic	0.90 (0.68-1.19)	1.08 (0.60-1.92)
Late pandemic	0.71 (0.49-1.05)	0.80 (0.39-1.64)

Abbreviation: OR, odds ratio.

^a^ Data were assessed using conditional logistic regression, and adjusted models account for age and parity.

## Discussion

This cohort study examined patterns in GWG across the first 2 years of the COVID-19 pandemic in Louisiana. Individuals who spent any part of part of their pregnancy, either delivery or conception, during the COVID-19 pandemic were less likely to gain within GWG recommendations compared with individuals before the pandemic. On average, individuals began pregnancy at a slightly higher weight, and changes in GWG patterns varied by prepregnancy BMI. For individuals with normal weight BMI before pregnancy, GWG decreased later into the pandemic. Yet in individuals with overweight and obesity, the mean GWG increased and remained high. There was no difference in GWG among the subsample of participants with multiple deliveries after accounting for time-varying factors.

The amount of total GWG after the pandemic in the current study (12.5-12.9 kg) is slightly lower than other samples (13.3 kg),^22^ likely owing to the high proportion of participants with obesity in the sample (33.4%), who gain less GWG.^22^ Many participants gained above GWG recommendations (43.0%-45.7%), which is comparable to racially and weight diverse prepandemic samples (47.2%),^1^ but not first-year pandemic samples (32%).^21^ These differences highlight the need for representative and diverse samples. Given that physical activity wanes into pregnancy,^33,34^ a proposed mechanism of lower GWG in late pandemic may be fewer restrictions (eg, closures) allowed for higher physical activity in early pregnancy along with healthier eating across pregnancy.^35,36^ Still, individuals who gave birth during partial and peak pandemic periods began pregnancy at a slightly higher weight (vs prepandemic participants) and gained more GWG during their pregnancy. Thus, more partial and peak pandemic individuals exceeded GWG recommendations for their heavier prepregnancy weight. This amount translates to approximately 0.82 kg per person on average but 1.40 to 2.40 kg per person for those with obesity, who gained greatly above guidelines throughout the pandemic. This is a small but clinically meaningful amount, because a 1-kg reduction can help pregnant individuals achieve GWG guidelines and improve health outcomes.^37^ These results emphasize the importance of considering both GWG and prepregnancy weight in understanding the full picture of a pregnancy.

The month-by-month analysis revealed that prepandemic conceptions had lower GWG on average, but few changes across the pandemic itself. The decrease in weight gain in March 2020 is unusual, though there were no differences in missing data. This finding may reflect fewer prenatal visits or health care utilization at the time of lockdown,^38,39^ so participants may have been less aware of their weight. Alternatively, variant surges may have caused additional precautions and restrictions even in later parts of the pandemic.^40^ The variable nature of the pandemic, accumulated weight gain, and outbreaks overshadowed any major month-to-month effects.

The within-person analysis revealed no major association of the pandemic after considering covariates.^24^ Those who had a second pregnancy during the pandemic may be more financially secure and report lower stress compared with others,^41^ and, thus, were less likely to gain above GWG recommendations in general. Even so, individuals tend to gain a similar amount of GWG in consecutive pregnancies,^42^ suggesting that the pandemic changed parts of the environment but not enough for major shifts in meeting GWG recommendations.^43^ In this sample, participants did have a slightly higher BMI, on average, to start their pandemic pregnancy, and there are negative associations of extra weight between pregnancies with pregnancy and infant outcomes.^44,45,46^

### Strengths and Limitations 

Strengths of the current study include a diverse sample, paired interpregnancy analysis to address confounding, and consideration of later COVID-19 pandemic periods. These opportunities allowed detailed study of GWG across an extended yet critical period in a diverse, southern US state.

This study is also not without limitations. First, self-report of weight may be subject to multiple biases, and we were unable to validate it in this study without a comparison, although these measures still correlate with anthropometric measures.^47^ Participants likely underestimate their weights; thus, total GWG may be higher.^48^ Second, although the current sample was representative of a metropolitan area,^49^ the results may not generalize to other geographic areas. Third, this study was limited to the first 2 years of the COVID-19 pandemic. The long-term outcomes of this global disaster are unclear.^50,51^ This investigation supports accounting for timing of the pandemic and the nuances of GWG guidelines based on prepregnancy BMI (ie, less GWG recommended because individuals begin pregnancy heavier).

## Conclusions

GWG was higher across the first 2 years of the COVID-19 pandemic compared with before the pandemic but approached prepandemic levels in later pandemic deliveries. Participants who delivered later in the pandemic gained less weight, on average, but began their pregnancy at a slightly higher weight. These findings suggest that subtle but clinically meaningful changes in prepregnancy weight and GWG are among the changes associated with the COVID-19 pandemic and that individuals with overweight and obesity were the most impacted.

## Notes

Supplement 1. **eTable 1.** 2009 Institute of Medicine Guidelines for Gestational Weight Gain by Prepregnancy Body Mass Index **eFigure 1.** Polynomial Model of Average Gestational Weight Gain by Month of Delivery, by Prepregnancy Weight **eFigure 2.** Polynomial Model of Average Gestational Weight Gain by Month of Conception, by Prepregnancy Weight **eTable 2.** Descriptive Statistics of Population With High Weight Gain by Period of Delivery Date (n=20,268) **eTable 3.** Descriptive Statistics of the Population With Prepandemic and Postpandemic Delivery by Gestational Weight Gain for Both Pregnancies

Supplement 2.
Data Sharing Statement
