# Supplementary material for: Trends in Gestational Weight Gain in Louisiana, March 2019 to March 2022
Source: JAMA Netw Open. 2023 Aug 29;6(8):e2331277. doi: 10.1001/jamanetworkopen.2023.31277 (PMC10466167; doi:10.1001/jamanetworkopen.2023.31277)
Supplement: Supplement 1. — eTable 1. 2009 Institute of Medicine Guidelines for Gestational Weight Gain by Prepregnancy Body Mass Index eFigure 1. Polynomial Model of Average Gestational Weight Gain by Month of Delivery, by Prepregnancy Weight eFigure 2. Polynomial Model of Average Gestational Weight Gain by Month of Conception, by Prepregnancy Weight eTable 2. Descriptive Statistics of Population With High Weight Gain by Period of Delivery Date (n=20,268) eTable 3. Descriptive Statistics of the Population With Prepandemic and Postpandemic Delivery by Gestational Weight Gain for Both Pregnancies [file jamanetwopen-e2331277-s001.pdf]

Supplemental Online Content

Harville EW, Kracht CL, Cohen NL, Sutton EF, Kebbe M, Redman LM. Trends in gestational weight gain in Louisiana, March 2019 to March 2022. *JAMA Netw Open*. 2023;6(8):e2331277. doi:10.1001/jamanetworkopen.2023.31277

eTable 1. 2009 Institute of Medicine Guidelines for Gestational Weight Gain by Prepregnancy Body Mass Index

eFigure 1. Polynomial Model of Average Gestational Weight Gain by Month of Delivery, by Prepregnancy Weight

eFigure 2. Polynomial Model of Average Gestational Weight Gain by Month of Conception, by Prepregnancy Weight

eTable 2. Descriptive Statistics of Population With High Weight Gain by Period of Delivery Date (n=20,268)

eTable 3. Descriptive Statistics of the Population With Prepandemic and Postpandemic Delivery by Gestational Weight Gain for Both Pregnancies

This supplemental material has been provided by the authors to give readers additional information about their work.

© 2023 Harville EW et al. *JAMA Network Open.*

eTable 1. 2009 Institute of Medicine Guidelines for Gestational Weight Gain by Prepregnancy Body Mass Index

BMI Category	Total GWG Range for singletons (kg)	Per 2nd and 3rd Trimester Mean (Range) (kg/week) for singletons	Total GWG Range for twins (kg)	Per 2nd and 3rd Trimester Mean (Range) (kg/week) for twins
<18.5 kg/m^2	12.5-18	0.51 (0.44 – 0.58)	N/A	N/A
18.5-24.9 kg/m^2	11.5-16	0.42 (0.35 –0.50)	16.8–24.5	0.73 (0.60 – 0.87)
25-29.9 kg/m^2	7-11.5	0.28 (0.23 –0.33)	14.1–22.7	0.65 (0.50, 0.81)
≥30 kg/m^2	5-9	0.22 (0.17 –0.27)	11.3–19.1	0.54 (0.40, 0.68)

^BMI, Body Mass Index; GWG, gestational weight gain: N/A = not applicable, as individual specific. These mothers were not included.

© 2023 Harville EW et al. *JAMA Network Open.*

eFigure 1. Polynomial Model of Average Gestational Weight Gain by Month of Delivery, by Prepregnancy Weight. Red line, March 2020 (start of pandemic); blue line, nine months after start of pandemic. p<0.05 for all.

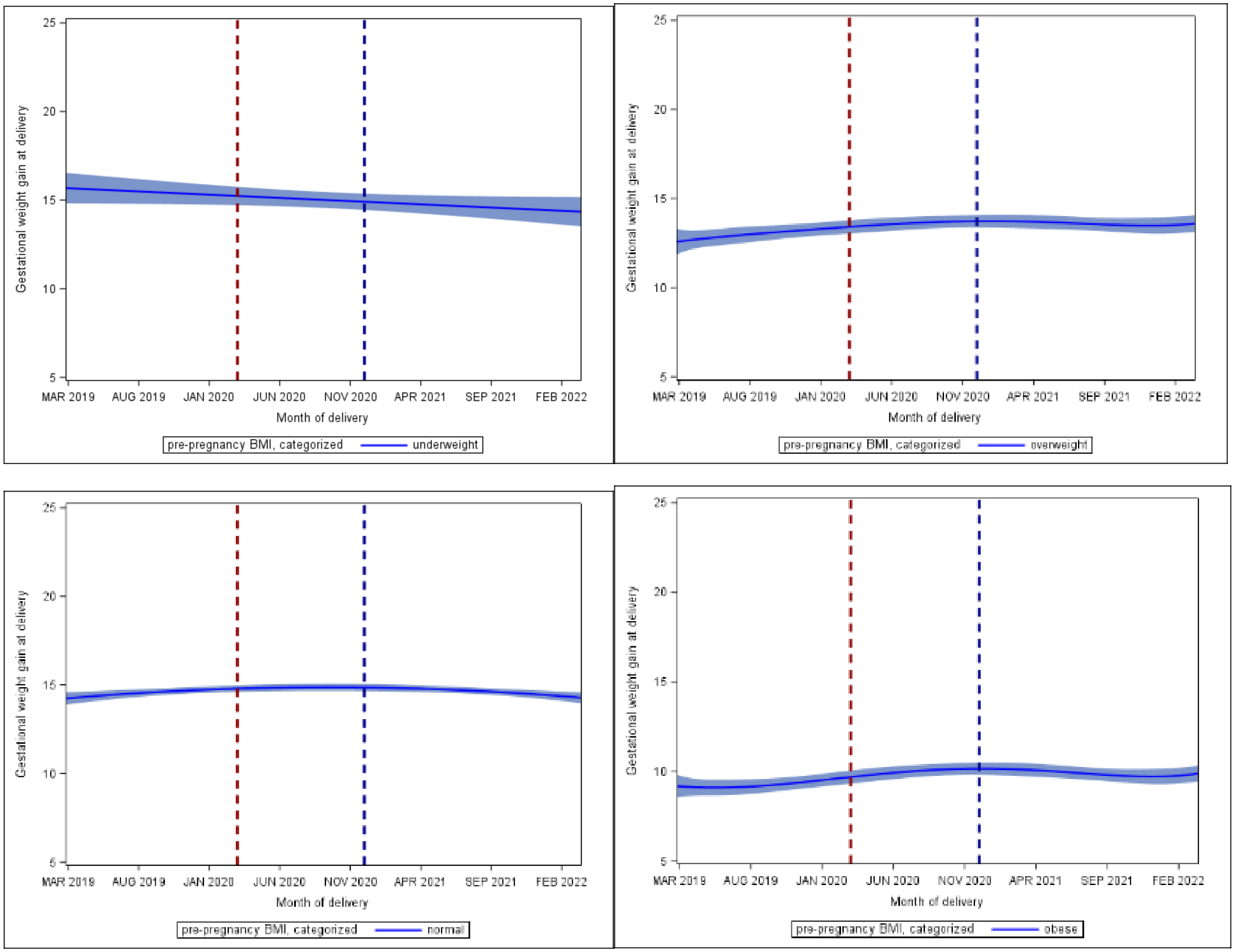

© 2023 Harville EW et al. *JAMA Network Open.*

eFigure 2. Polynomial Model of Average Gestational Weight Gain by Month of Conception, by Prepregnancy Weight. Red line, March 2020 (start of pandemic); blue line, nine months before start of pandemic. $p<0.05$ for all except underweight BMI category.

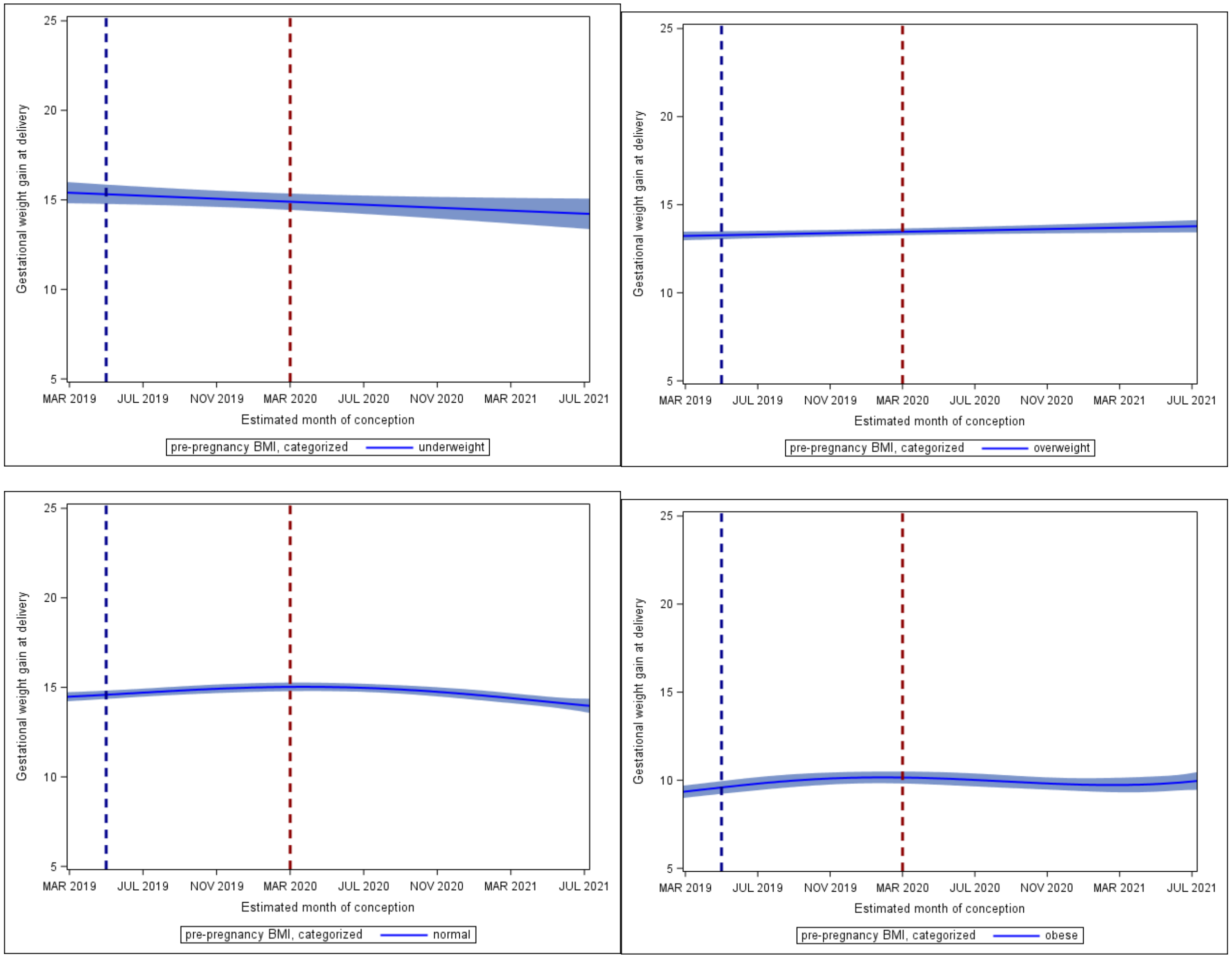

© 2023 Harville EW et al. *JAMA Network Open.*

eTable 2. Descriptive Statistics of Population With High Weight Gain by Period of Delivery Date (n=20,268)^

	Pre-Pandemic		Peak Pandemic		Late Pandemic		
Dates	3/13/2019 – 3/12/2020		3/13/2020 – 3/12/2021		3/13/2021 – 3/12/2022		
Total Sample	7408		6899		5961		
	Mean ± SD	(min-max)	Mean ± SD	(min-max)	Mean ± SD	(min-max)	p-value
**Total GWG (kg)**							
Overall	12.47 ± 7.64	(-9.07-45.36)	13.04 ± 7.60	(-9.07-45.36)	13.02 ± 8.04	(-9.07-45.36)	<.001
Stratified by BMI							
<18.5 kg/m^2	14.98 ± 6.02	(1.81-38.56)	15.59 ± 6.36	(3.63-41.73)	14.63 ± 5.76	(-1.36-44.91)	0.30
18.5-24.9 kg/m^2	14.59 ± 6.41	(-5.90-45.36)	14.84 ± 6.40	(-5.44-44.91)	14.93 ± 6.82	(-4.99-45.36)	0.15
25-29.9 kg/m^2	13.07 ± 7.60	(-6.80-44.91)	13.87 ± 7.42	(-7.71-45.36)	13.95 ± 7.86	(-7.26-45.36)	0.001
≥30 kg/m^2	9.29 ± 8.05	(-9.07-45.36)	10.01 ± 8.20	(-9.07-42.64)	10.11 ± 8.71	(-9.07-44.91)	0.001
**Total GWG over IOM Recs (kg)**							
Overall	6.22 ± 5.46	(0.07-36.36)	6.32 ± 5.53	(0.07-33.86)	6.85 ± 5.89	(0.07-35.91)	<.001
Stratified by BMI							
<18.5 kg/m^2	5.40 ± 4.10	(0.14-20.56)	4.21 ± 4.84	(0.14-23.73)	4.54 ± 5.08	(0.14-26.91)	0.39
18.5-24.9 kg/m^2	5.15 ± 4.71	(0.33-29.36)	5.14 ± 4.76	(0.33-28.91)	5.65 ± 4.94	(0.33-29.36)	0.04
25-29.9 kg/m^2	6.78 ± 5.47	(0.29-32.50)	6.78 ± 5.53	(0.29-33.86)	7.29 ± 5.76	(0.29-33.86)	0.08
≥30 kg/m^2	6.69 ± 5.96	(0.07-36.36)	7.02 ± 5.98	(0.07-32.28)	7.52 ± 6.54	(0.07-35.91)	0.01
	n (%)		n (%)		n (%)		
**200% above IOM Recs**							
Overall	531 (7.2)		553 (8.0)		554 (9.3)		<.001
Stratified by BMI							
<18.5 kg/m^2	1 (0.5)		3 (1.5)		1 (0.6)		0.46
18.5-24.9 kg/m^2	41 (1.4)		36 (1.4)		37 (1.7)		0.64
25-29.9 kg/m^2	173 (9.3)		176 (9.8)		171 (11.4)		0.12
≥30 kg/m^2	316 (13.0)		338 (15.2)		345 (16.9)		0.001
**Top 10% GWG per BMI**							
Overall	736 (9.9)		743 (10.8)		712 (11.9)		0.001
Stratified by BMI							
<18.5 kg/m^2	30 (14.2)		22 (11.2)		13 (7.2)		0.08
18.5-24.9 kg/m^2	304 (10.5)		297 (11.1)		283 (12.7)		0.046
25-29.9 kg/m^2	177 (9.5)		181 (10.0)		176 (11.7)		0.10
≥30 kg/m^2	225 (9.2)		243 (10.9)		240 (11.7)		0.02

© 2023 Harville EW et al. *JAMA Network Open.*

^ 200% over IOM recommendations are calculated by total GWG divided by the IOM maximum recommendation per pre-pregnancy BMI group greater than or equal to 2. For multiple births, the maximum recommendations are: 28.1 kg (underweight), 24.5 kg (normal), 22.7 kg (overweight), 19.1 kg (obese). For singletons, the maximum recommendations are: 18 kg (underweight), 16 kg (normal), 11.5 kg (overweight), 9 kg (obese). The cutoff points for top 10% GWG per pre-pregnancy BMI group are 22.68 kg (underweight), 22.68 kg (normal), 23.13 kg (overweight), 20.41 kg (obese). adjusted for maternal age, parity, race/ethnicity, education, marital status; analysis was limited to the first pregnancy in the dataset per woman.

© 2023 Harville EW et al. *JAMA Network Open.*

eTable 3. Descriptive Statistics of the Population With Prepandemic and Postpandemic Delivery by Gestational Weight Gain for Both Pregnancies^

	Pre: Above<br>Post: Recommended/Under		Pre: Recommended/Under<br>Post: Above		Pre: Recommended/Under<br>Post: Recommended/Under		Pre: Above<br>Post: Above		Discrepant only	Overall
	Mean ± SD	n (%)	Mean ± SD	n (%)	Mean ± SD	n (%)	Mean ± SD	n (%)	p-value	p-value
Age (years)	28.8 ± 4.4		29.2 ± 4.9		29.4 ± 5.0		29.6 ± 4.8		0.38	0.23
Interpregnancy Interval (years)	1.87 ± 0.59		1.89 ± 0.56		1.90 ± 0.56		1.88 ± 0.58		0.77	0.91
Age at 2nd pregnancy (years)									0.12	0.08
<18		0 (0)		1 (0.6)		1 (0.2)		0 (0)		
18-25		42 (20.0)		31 (18.5)		117 (19.9)		54 (17.0)		
25-30		76 (36.2)		53 (31.6)		175 (29.7)		94 (29.2)		
30-35		74 (35.2)		53 (31.6)		200 (34.0)		122 (37.9)		
35-40		18 (8.6)		28 (16.7)		82 (13.9)		47 (14.6)		
>40		0 (0)		1 (0.6)		14 (2.2)		5 (1.6)		
Race/Ethnicity									0.66	0.27
Asian		3 (1.5)		1 (0.6)		15 (2.6)		9 (2.8)		
Black		81 (38.8)		66 (39.5)		235 (40.5)		109 (34.3)		
Hispanic		7 (3.4)		9 (5.4)		32 (5.5)		11 (3.5)		
White		118 (56.5)		91 (54.5)		298 (51.4)		189 (59.4)		
Marital Status									0.41	0.06
Married		111 (52.9)		96 (57.1)		327 (55.5)		204 (63.4)		
Not Married		99 (47.1)		72 (42.9)		262 (44.5)		118 (36.7)		
Insurance									0.86	0.03*
Private		111 (52.4)		89 (53.3)		279 (47.4)		185 (57.5)		
Medicaid/other		99 (47.6)		78 (46.7)		310 (52.6)		137 (42.6)		
Smoking									0.67	0.20
Nonsmoker		193 (92.4)		156 (92.9)		537 (92.0)		299(93.2)		
Former		7 (3.4)		7 (4.2)		26 (4.5)		19 (5.9)		
Current		9 (4.3)		5 (3.0)		21 (3.6)		3 (0.9)		
Body Mass Index (kg/m^2)										
Pre-Pandemic Pregnancy	27.4	6.9		30.5	8.0		26.7		<0.01*	<0.01*
Post Pandemic Pregnancy	30.7	8.4		29.2	7.3		27.4		0.06	<0.01*

© 2023 Harville EW et al. *JAMA Network Open.*

*^Categories created are referring to gaining above ("above)", within ("recommended") or under ("under") gestational weight gain guidelines for their pre-pregnancy body mass index; assessed using chi-square or Fisher exact test for categorical outcomes or One way analysis of variance for continuous outcomes, $p<0.05$**

© 2023 Harville EW et al. *JAMA Network Open.*
