# Supplementary material for: Trends in Gestational Weight Gain in Louisiana, March 2019 to March 2022
Source: JAMA Netw Open. 2023 Aug 29;6(8):e2331277. doi: 10.1001/jamanetworkopen.2023.31277 (PMC10466167; doi:10.1001/jamanetworkopen.2023.31277)
Supplement: Supplement 2. — Data Sharing Statement [file jamanetwopen-e2331277-s002.pdf]

Data Sharing Statement

Harville. Trends in Gestational Weight Gain in Louisiana, March 2019 to March 2022. *JAMA Netw Open*. Published August 29, 2023. doi:10.1001/jamanetworkopen.2023.31277

Data
Data available: No

Additional Information
Explanation for why data not available: This is electronic health records. We will make a de-identified database available upon request.
